# Supplementary material for: From sequence to enzyme mechanism using multi-label machine learning
Source: BMC Bioinformatics. 2014 May 19;15:150. doi: 10.1186/1471-2105-15-150 (PMC4229970; doi:10.1186/1471-2105-15-150)
Supplement: Additional file 2 — Java code of ml2db. Additional file ml2db_code.tar.gz contains the Java source code to run the multi-label machine learning experiments and save the results to database. The code’s Javadoc is included. [file 1471-2105-15-150-S2.zip › additional file 2/ml2db/utils/doc/uk/ac/ed/inf/utils/collectionutils/setutils/class-use/Set.html]

Uses of Class uk.ac.ed.inf.utils.collectionutils.setutils.Set


---


|  |  |  |
| --- | --- | --- |
| **Overview** \| **Package** \| **Class** \| **Use** \| **Tree** \| **Deprecated** \| **Index** \| **Help** | |  |
| PREV   NEXT | **FRAMES**    **NO FRAMES**     **All Classes** |  |


---


## **Uses of Class uk.ac.ed.inf.utils.collectionutils.setutils.Set**

| Packages that use Set | |
| --- | --- |
| **test.setutils** |  |
| **uk.ac.ed.inf.utils.collectionutils.setutils** |  |

Uses of Set in test.setutils

| Methods in test.setutils that return Set | |
| --- | --- |
| `static Set` | `SetTest.getSet(java.lang.String id, java.util.TreeSet<java.lang.String> elements)` |
| `static Set` | `SetTest.getSetA()` |
| `static Set` | `SetTest.getSetAB()` |
| `static Set` | `SetTest.getSetABC()` |
| `static Set` | `SetTest.getSetAC()` |
| `static Set` | `SetTest.getSetAD()` |

Uses of Set in uk.ac.ed.inf.utils.collectionutils.setutils

| Methods in uk.ac.ed.inf.utils.collectionutils.setutils that return Set | |
| --- | --- |
| `Set` | `Set.clone()` |

| Methods in uk.ac.ed.inf.utils.collectionutils.setutils that return types with arguments of type Set | |
| --- | --- |
| `java.util.TreeSet<Set>` | `SupersetsManager.createSupersets()` |
| `java.util.TreeSet<Set>` | `SupersetsManager.getSets()` |
| `java.util.TreeSet<Set>` | `Set.getSubsets()` |
| `java.util.TreeSet<Set>` | `SupersetsManager.getSupersets()` |

| Methods in uk.ac.ed.inf.utils.collectionutils.setutils with parameters of type Set | |
| --- | --- |
| `void` | `Set.addSubset(Set newsetToAdd)` |
| `int` | `Set.compareTo(Set otherSet)` |
| `boolean` | `Set.contains(Set set)` |

Constructor parameters in uk.ac.ed.inf.utils.collectionutils.setutils with type arguments of type Set

`Set(java.lang.String id,
java.util.TreeSet<java.lang.String> elements,
SupersetsManager manager,
java.util.TreeSet<Set> subsets)`

---


|  |  |  |
| --- | --- | --- |
| **Overview** \| **Package** \| **Class** \| **Use** \| **Tree** \| **Deprecated** \| **Index** \| **Help** | |  |
| PREV   NEXT | **FRAMES**    **NO FRAMES**     **All Classes** |  |


---
